# Extraction Optimization, Characterization and Bioactivities of a Major Polysaccharide from *Sargassum thunbergii*

**DOI:** 10.1371/journal.pone.0144773

**Published:** 2015-12-09

**Authors:** Xiumei Yuan, Yawei Zeng, Kaiying Nie, Dianhui Luo, Zhaojing Wang

**Affiliations:** Department of Bioengineering and Biotechnology, Huaqiao University, Fujian Xiamen, China; Islamic Azad University-Mashhad Branch, Mashhad, Iran, IRAN, ISLAMIC REPUBLIC OF

## Abstract

*Sargassum thunbergii* is a kind of natural edible algae. STP (*S*. *thunbergii* polysaccharides) was considered as the main bioactive compounds in *S*. *thunbergii*. To obtain the optimal processing conditions for maximum total sugar yield, **s**ingle factor investigation and response surface methodology (RSM) were employed. The optimal processing conditions were as follows: liquid to solid ratio 120 mL/g, extraction time 210 min, extraction temperature 97°C. The experimental yield 7.53% under optimized conditions was closely agreed with the predicted yield 7.85% of the model. The major polysaccharide fraction from *S*. *thunbergii* (named STP-II) was purified by DEAE-Sepharose CL-6B column chromatography. High-performance size-exclusion chromatography (HPSEC), gas chromatography (GC) and high-performance liquid chromatography (HPLC) were used to identify its characterizations, and in vitro antioxidant assays and cytotoxicity assays were used to research its bioactivities. The purified fraction STP-II (63.75%) was a single peak in HPSEC with Sugar KS-804 column, had a molecular weight of 550KD, and comprised mainly of fucose, xylose, galactose, glucose and glucuronic acid. STP-II had higher scavenging activities on hydroxyl radical (76.72% at 0.7 mg/mL) and superoxide radical (95.17% at 2 mg/mL) than Vitamin C (Vc). STP-II also exhibited the capability of anti-proliferation in Caco-2 cells. STP-II possessed good antioxidant and inhibitory activity against human colon cancer Caco-2 cells in vitro and could be explored as novel natural functional food.

## Introduction

*Sargassum thunbergii* is a kind of natural economic algae, and it is the dominant species of algae community in summer, growing on the rocks of low tide belt [1–2]. *S*. *thunbergii* has been recorded on “China's Marine medicine dictionary” for its function of resolving hard lump and diuresis detumescence effect. The crude polysaccharide extracted from *S*. *thunbergii* had many biological activities such as antioxidant activity [3], hpyerglycemic effect [4], anti-inflammatory activity [5–6], anti-coagulation activity, and anticancer effect [7]. These activities are very valuable for natural drug development, and this extract has thus attracted much attention and research. Several reports about the monosaccharide composition of polysaccharides from *S*. *thunbergii* have been published [5]. However, there is no much studying on the extraction optimization and detailed characterization and bioactivities of purified polysaccharides from *S*. *thunbergii*. This situation significantly restricts the further development of *S*. *thunbergii* in other fields, including the energy, medical and food industries.

The objective of this study was to optimize the conditions for total sugars extraction from *S*. *thunbergii*. Based on single factor investigation, response surface methodology [8–10], and a three-levels and three variables central composite rotatable design, we studied the effects of the ratio of liquid to solid, extraction time and extraction temperature on the yield of total sugars, and obtained optimum conditions for the maximum production. In addition, the major polysaccharide from *S*. *thunbergii* was purified, and its characterization and bioactivities were identified.

## Materials and Methods

### Materials and Chemicals

*S*. *thunbergii* was purchased from a local store (Dalian, Liaoning Province, China). The material was identified by Professor Liu, Huaqiao University, Fujian, China. Monosaccharide and dextran standard samples (Sigma Co., St Louis, MO, USA), Nitro Blue Tetrazolium (NBT, Amresco Inc., Solon, OH, USA), Nicotinamide Adenine Dinucleotide Hydrogen (NADH, Amresco Inc., Solon, OH, USA), and Phenazine Methosulfate (PMS, Fluka Inc., St Louis, MO, USA) were purchased from a local agent (Taijing Co., Xiamen), Thiazolyl Blue Tetrazolium Bromide (MTT, Sigma Co., St Louis, MO, USA), while DEAE Sepharose CL-6B was acquired from Pharmacia Co. (Sweden). All other reagents used were of analytical grade.

### Extraction of Total Sugar from *S*. *thunbergii*

Dry *S*. *thunbergii* powder (1 g) was used for each treatment. The juice was extracted by heating *S*. *thunbergii* powder with a certain volume of distill water (pH = 2.0, adjusted by HCl) at a selected temperature for a period of time. The supernatant was collected by the centrifuge for the determination of total sugar yield.

### Determination of the Yield

Total sugar concentration was measured by phenol-sulfuric acid method [11]. The total sugar yield (%, w/w) was calculated as the total sugar content of extraction divided by dried sample weight.

### Experimental Design and Statistical Analysis

Firstly, a single factor investigation was used to study the effects of the key factors namely liquid:solid ratio (mL/g), extraction time (min), and extraction temperature (°C) on the yield of total sugar. By changing one factor while other factors kept invariable, we could get the rule of the total sugar yield changing with the variable factor.

After determining the preliminary range of extraction variables according to single-factor test, we optimized the process by Design-Expert (Version 9.0.3.1, Stat-Ease Inc., Minneapolis, MN, USA) software, and a three-level-three-factor, Box–Behnken design (BBD) was employed in this optimization system [12–13]. Liquid to solid ratio(A), extraction time (B) and extraction temperature(C) were the independent variables, and the total sugar yield (Y) was the response function to the independent variables. According to Box–Behnken factorial design, the complete design considered as 17 experimental points including five replications of the center points.

Three factors chosen were designated as A, B, and C. Each of them was divided into three levels, and coded +1, 0, −1 for high, intermediate and low value, respectively. The fitted polynomial equation was expressed as surface and contour plots for visualizing the relationship between the response and experimental levels of each factor and deducing the optimum conditions.

### Purification of Major Polysaccharide

Dry *S*. *thunbergii* powder (100 g) was extracted with 1.2×10^4^ mL of distilled water (pH = 2.0, adjusted by HCl) at 97°C for 3.5 h once. The extracts were then concentrated and submitted to graded precipitation with four volumes of ethanol, and the mixture was kept overnight at 4°C to precipitate the polysaccharides. The precipitate was collected by centrifugation, and dried in a freeze drier to produce crude polysaccharides. The crude polysaccharide mix (500 mg) was dissolved in 10 mL distilled water and centrifuged. The supernatant was then injected into a column (4.6 cm×40 cm) of DEAE-Sepharose CL-6B equilibrated with three column volumes with distilled water. After loading with sample, the column was eluted with distilled water at 50 mL/h (12 min/tube), followed by NaCl aqueous solution (0 to 1 M) for 500 mL. The major polysaccharide fractions were collected with a fraction collector from the NaCl aqueous solution, and dialyzed against tap water and distilled water for 48 h. The dialyzed solution was collected by centrifugation and dried in a freeze drier to produce a major purified polysaccharide.

### Purity and Molecular Weight Determination

The purity and molecular weight (MW) of the polysaccharide were determined by high-performance size-exclusion chromatography (HPSEC) (1100 system, Agilent Technologies, Palo Alto, CA, USA) with a gel-filtration chromatographic column of Shodex Sugar KS-804 (Showa Denko K.K, Japan) and a refractive index detector (RID). The purified polysaccharide (10 mg) was dissolved in distilled water (1 mL) and passed through a 0.22-μm filter. It was then applied to a gel-filtration chromatographic column, maintained at a temperature of 50°C, eluted with distilled water at a flow rate of 1.0 mL/min and detected by RID. The molecular weight was calculated by a calibration curve obtained by using various standard dextrans of different molecular weights (Dextran Blue, Dextran T10, T40, T70, T500 and Glucose) [14]. Total content of carbohydrate was determined by phenol-sulfuric acid method [11]. The concentration of proteins was measured according to Bradford's method [15]. Total uronic acid was determined using the sulfuric acid carbazole method [16].

### Monosaccharide Composition Analysis

Gas chromatography (GC) and high-performance liquid chromatography (HPLC) were used for the identification of monosaccharide composition. For GC analysis, the sample (10 mg) was dissolved in 1 mL of a 1 M HCl-Methanol (w/w = 1/21), and derivation was then carried out using the trimethylsilylation reagent. The monosaccharide compositions were analyzed on a gas chromatography system (6890 system, Agilent Technologies, Palo Alto, CA, USA) equipped with an HP-5 column (30 m×0.25 mm×0.25 μm) and a flame-ionization detector (FID). For HPLC analysis, the purified polysaccharide (100 mg) was hydrolyzed with 1 M H_2_SO_4_, kept at 100°C for 8 h. The content was neutralized to pH 7.0 with barium carbonate, filtrated, concentrated and freeze-dried. It was then applied to a gel-filtration chromatographic column of Shodex Sugar SP0810 (Showa Denko K.K, Japan), maintained at a temperature of 80°C, eluted with distilled water at a flow rate of 0.5 mL/min and detected by a refractive index detector (RID) [17].

### Fourier Transform Infrared Spectroscopy (FTIR) Analysis

The FTIR spectrum of the polysaccharide was determined using a Fourier Transform Infrared Spectrometer (Nicolet iS10, Thermo Fisher Scientific, Waltham, MA, USA). The purified polysaccharide was ground with KBr powder and then pressed into pellets for FTIR measurement at a frequency range of 4000~400 cm^-1^.

### Cell Culture and Cytotoxicity Assay

Caco-2 cells were cultured in DMEM medium (high glucose) in a cell incubator with 5% CO_2_ at 37°C. Experiments were performed when cell growth was approximately 80% confluent. Three independent experiments were performed. The cytotoxicity of STP-II against the cancer cell line was determined using the MTT assay. Briefly, cancer cells in logarithmic phase (5×10^5^/mL) were seeded in 96-well culture dishes at a dose 100 μl/well. After 12–24 h, the cells were treated with 100 μl DMEM medium of sample (final concentrations of 0.2, 0.3, 0.6, 2.5, and 5g/mL). Cis-platin were used as the positive control. After treatment of 36 h, 20 μL of MTT (5 mg/mL) was added to each well and the cells were incubated for another 4 h at 37°C. After the media were removed, 150 μL of DMSO was added to each well to dissolve the cellular crystalline deposits and the absorbance was determined at 570 nm. The inhibition rate (I %) was calculated according to the formula below [18].

$$Inhibition\;rate\;\left(I\;\%\right)\;=A_{570nm,control}-A_{570nm,sample}\;/\;A_{570nm,contol}-A_{570nm,blank}$$

### Antioxidant Activities Assay

The superoxide radical assay and the hydroxyl radical assay were measured by the methods of Ghiselli and Robak with a minor modification, respectively [19].

The scavenging ability for DPPH of sample was investigated. Briefly, samples or vitamin C (Vc) were dissolved in distilled water at 0, 0.5, 1.25, 2.5, 5, 10, 20 and 40 mg/mL. The sample solution (0.1 mL) or distilled water (0.1 mL, control) was mixed with 2 mL of 0.3 mM DPPH, was incubated for 20 min at 25°C. The absorbance of the mixture was measured at 517 nm. The scavenging ability for DPPH of sample was calculated using the equation (1 –absorbance of sample/absorbance of control) × 100%.

## Results and Discussion

### Single Factor Results

In order to study the effects of different liquid:solid ratio on the yield, we selected 20:1, 40:1, 60:1, 80:1, 100:1, 120:1, 140:1 liquid:solid ratio sequentially, while the extraction time was 60 min, and the extraction temperature was 80°C. The result showed that the yield increased as the liquid:solid ratio ascended, and after the liquid:solid ratio reached 100:1, the yield was almost invariable. According to the literature [20], if the ratio of liquid to solid was too small, polysaccharides in raw material could not be completely extracted up, on the contrary it would cause high process cost. So it was better that liquid:solid ratio was chose between 80:1 and 120:1 in latter experiments.

Extraction time is a factor that would influence the extraction efficiency and selectivity of the fluid [21–22]. The effect of extraction time on the yields had been studied under the reaction conditions as follows: liquid:solid ratio 100:1, extraction temperature 80°C, and the extraction time adopted 20 min, 60 min, 100 min, 140 min, 180 min, 220 min and 260 min. The results showed the yield was always enhanced before 180 min, and then it was reached the highest and almost not changing with the extraction time adding.

Temperature is an important factor in the extraction of polysaccharides, the raise of the polysaccharides diffusion coefficient and the increased solubility of the polysaccharides in the extracting solvent at higher temperatures caused the increase of the polysaccharides mass going out from the algae into the solution [23]. This experiment in turn adopted 30°C, 50°C, 75°C and 100°C to investigate the effects of extraction temperature on the yield. The other two factors remained invariable: liquid:solid ratio 100:1, extraction time 180 min. The yield increased gradually with elevating temperature. Even though the yield may be higher if the temperature elevated after 100°C, we would not continue the work. Due to the polysaccharide were extracted in water bath, the temperature could not exceed 100°C at room temperature and atmosphere pressure. On the other hand, the polysaccharide could resolve if the temperature was too high. It was a complex and costly exercise with no guarantee of success to elevate temperature beyond 100°C.

### Optimization of the Extraction Parameters

According to Box–Behnken factorial design, the results of 17 experiments were shown in Table 1. A Design-Expert Software was employed to generate the statistical analysis and regression model. The regression equation was given in the following:

$$Y=4.92+0.48A+0.59B+1.64C+0.23AB+0.65AC+0.52BC-0.99A^{2}+0.57B^{2}-1.72C^{2}.$$

Where Y was the predicted yield of total sugar, and A, B and C were the coded values for liquid:solid ratio, extraction time and extraction temperature, respectively.

**Table 1:** Box–Behnken experimental design and results for the yield of total sugar.

Run	Coded variable levels			Yield (%)
	A (mL/g)	B (min)	C (°C)	
**1**	-1(80)	-1(150)	0(85)	4.578
**2**	1(120)	-1(150)	0(85)	5.296
**3**	-1(80)	1(210)	0(85)	5.026
**4**	1(120)	1(210)	0(85)	6.67
**5**	-1(80)	0(180)	-1(70)	1.955
**6**	1(120)	0(180)	-1(70)	1.42
**7**	-1(80)	0(180)	1(100)	3.505
**8**	1(120)	0(180)	1(100)	5.555
**9**	0(100)	-1(150)	-1(70)	1.714
**10**	0(100)	1(210)	-1(70)	2.126
**11**	0(100)	-1(150)	1(100)	4.378
**10**	0(100)	1(210)	1(100)	6.887
**13**	0(100)	0(180)	0(85)	4.901
**14**	0(100)	0(180)	0(85)	4.102
**15**	0(100)	0(180)	0(85)	4.763
**16**	0(100)	0(180)	0(85)	5.406
**17**	0(100)	0(180)	0(85)	5.447

The statistical analysis showed that the proposed model was suitable, with significant p-value (p = 0.0005 < 0.01) and very satisfactory value of the R^2^ (0.9589) for the response. The significance of each coefficient was estimated by the Student’s F-test and p-value in Table 2.

**Table 2:** Test results of significance for regression coefficient.

Variables	Sum of squares	Mean square	*F*- value	*p*-Value
**A**	1.88	1.88	7.20	0.0314
**B**	2.81	2.81	10.77	0.0134
**C**	21.48	21.48	82.31	<0.0001
**AB**	0.21	0.21	0.82	0.3949
**AC**	1.67	1.67	6.40	0.03949
**BC**	1.10	1.10	4.21	0.0793
**A** ^**2**^	0.042	0.042	0.16	0.7016
**B** ^**2**^	1.36	1.36	5.21	0.0565
**C** ^**2**^	12.39	12.39	47.48	0.0002

The corresponding variables will be more significant if the absolute F-value becomes larger and the *P*-value becomes smaller [24]. It could be seen that significant terms were the linear terms of liquid:solid ratio (A), water extraction time (B), extraction temperature (C), the quadratic terms of extraction time (B^2^), extraction temperature (C^2^), as well as the interaction terms of liquid:solid ratio (A) and extraction temperature (C), the interaction terms of extraction time (B), and water extraction temperature (C). The linear terms of extraction temperature (*P*<0.0001) and the quadratic terms of extraction temperature (*P*<0.0002) had the largest effects on the yield of total sugar.

The "Lack of Fit" of 0.68 implied that it was not significant relative to the pure error. The difference of value between Adj R-Squared (0.9061) and Pred R-Squared (0.7347) was less than 0.2, suggesting that the regression model was adequate for prediction within the range of experimental variables [25].

The response surfaces and the contour plots which were the graphical representations of the regression model were obtained by Design-Expert. The normal plot of residuals and the map of residuals vs. predicted given by Design-Expert showed that the former was linear and the latter ruleless, which indicated that the model was credible. The tortuose surface and oval contour plot meant the dramatically complex interaction effects of liquid:solid ratio (A) and extraction temperature (C) [25].

### Verification Experiments

By Design-Expert, optimum conditions and predicting optimum response values were obtained. The calculated yield was 7.85% under predicted optimum conditions (A = 120 mL/g, B = 210 min, C = 97.29°C), and it was closely in agreement with the three experimental mean values of 7.53% obtained in the modified conditions (A = 120 mL/g, B = 210 min, C = 97°C). This result suggested that the model was satisfactory and accurate for the extraction process.

### Purification of Polysaccharide from *S*. *thunbergii*

**Fig 1:**
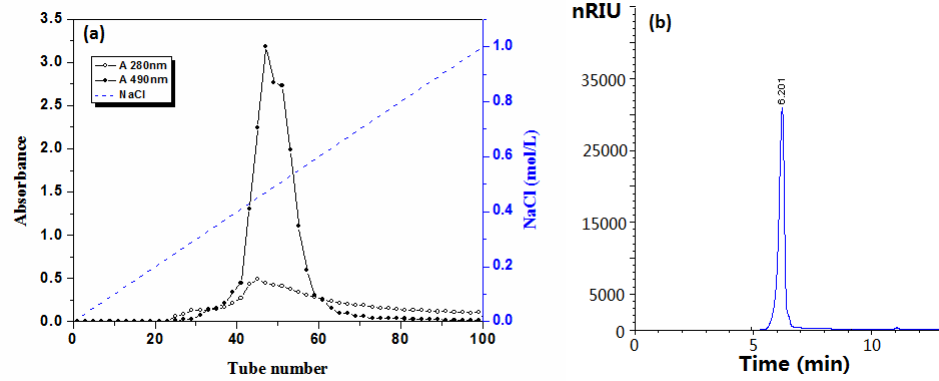
(a) Sugar and protein curves of STP-II from NaCl lined stepwise elute of DEAE Sepharose CL-6B column chromatogram and (b) HPSEC chromatogram of STP-II.

The dry powder of *S*. *thunbergii* (100 g) was extracted with optimum conditions, and the filtrate were concentrated and precipitated with ethanol. The precipitate was collected by centrifugation, and dried at a freeze drier, giving a crude polysaccharide STP by a yield of 20%. STP on fractionation by DEAE-Sepharose CL-6B (Fig 1A) gave a major polysaccharide fraction by a yield of 63.75%, named STP-II. The fraction was collected, dialyzed to remove impurities including pigments and free proteins [26], and freeze-dried. We came to a conclusion that STP-II was homogeneous by the following test. It was showed only one symmetrical peak on HPSEC with Sugar KS-804 column (Fig 1B), indicating that no other components were present in the sample. STP-II could contain the trace binding protein, because the tube number of elute curve of polysaccharide was the same as that of protein (Fig 1A) and STP-II had the good homogeneity by HPSEC (Fig 1B) [27].

### Physicochemical Properties of STP-II

**Fig 2:**
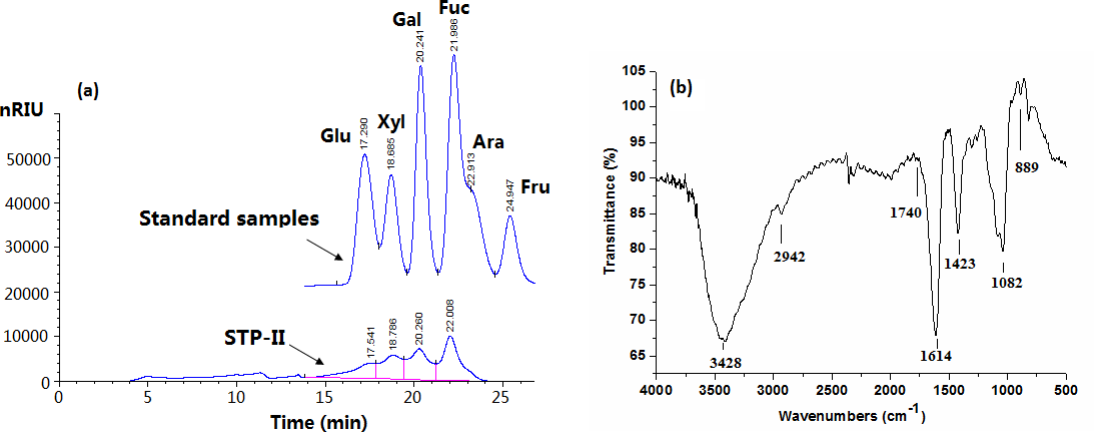
(a) HPLC chromatogram of standard samples and hydrolyzed STP-II and (b) IR spectra of STP-II from *S*. *thunbergii* (Fuc: fucose; Xyl: xylose; Gal: galactose; Glu: glucose).

The average molecular weight of STP-II was estimated from a calibration curve prepared with standard dextrans, and it was nearly 550KD. On hydrolysis by 1 M H_2_SO_4_, the presence of fucose, xylose, galactose and glucose were detected by HPLC analysis (Fig 2A) according to the retention time of standard monosaccharide samples. By GC analysis, the presence of fucose, xylose, galactose and glucose were verified, and glucuronic acid was detected. STP-II was composed of approximately 96.78% (w/w) of total carbohydrate, which contained about 6.83% uronic acid, and STP-II showed a trace response to the Bradford test.

The infrared spectrum of STP-II displayed a broad stretching intense characteristic peak at approximately 3428 cm^-1^ for the hydroxyl group, a weak C–H stretching band at approximately 2942 cm^-1^, and other characteristic absorption peaks of the polysaccharide at approximately 1614 cm^-1^, 1423 cm^-1^, and 1082 cm^-1^ (Fig 2B). The absorption peaks at 889±4 cm^-1^ confirmed the existence of β-glycosidic bond. The bond around 1740 cm^-1^ was presented, confirming that STP-II contained uronic acid [18].

### Antioxidant Activities

On the basis of DPPH radical assay, superoxide radical assay and hydroxyl radical assay, antioxidant activities of STP-II were investigated.

**Fig 3:**
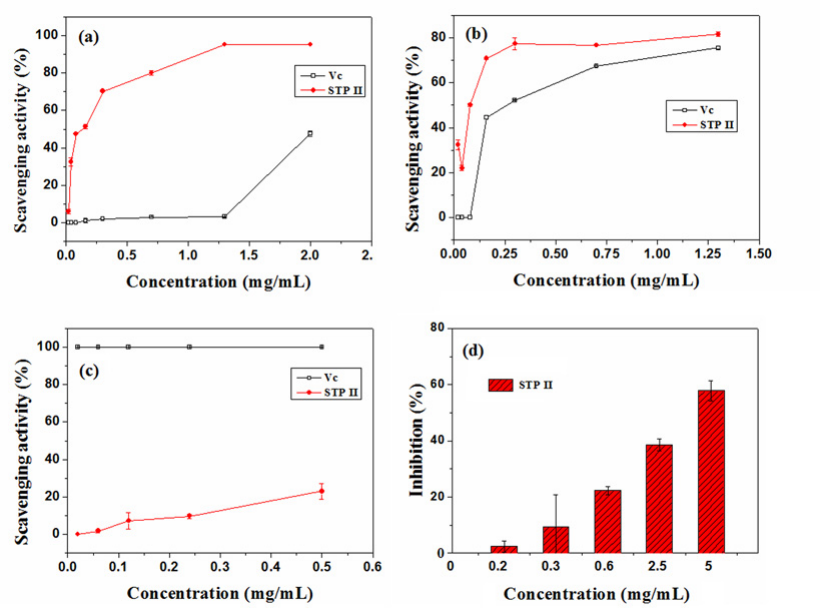
Bioactivities of STP-II: scavenging effects on (a) superoxide radical; (b) hydroxyl radical; (c) DPPH radical; (d) inhibitory effects on colon cancer cell growth.

Superoxide anion shows as a relatively weak oxidant, but it can cause damage to the cells and DNA leading to various diseases [28]. The result of scavenging activities of Vc and STP-II on superoxide radical was presented in Fig 3A. It showed that the scavenging activity of STP-II was higher than Vc, scavenging activity for superoxide anion of STP-II was 95.17% at final concentration of 2 mg/mL, and that of Vc 47.67% at final concentration of 2 mg/mL.

Hydroxyl radicals are well known as the most reactive free radicals, and they can react with almost all the bio-macromolecules functioning in living cells and induce severe damage to the adjacent bio-molecules [29]. The scavenging effects of Vc and STP-II on hydroxyl radicals were shown in Fig 3B, at final concentration of 0.02 to 0.7 mg/mL, the scavenging activity of STP-II was from 32.42% to 76.72%, which was higher than Vc [30].

The DPPH free radical is a stable compound, which has been widely used to evaluate the free radical scavenging ability of various samples. The scavenging activities of Vc and STP-II on DPPH radical were showed as Fig 3C, the scavenging activity of STP-II significantly increased with the increasing concentrations, and it indicated that Vc had a higher scavenging effect than STP-II.

### Cytotoxicity of STP-II against Caco-2 Cells

The cytotoxicity induced by STP-II was investigated in human colon cancer cell line Caco-2 using the MTT assay. As shown in Fig 3D, STP-II exhibited the capability of anti-proliferation in Caco-2 cells, with the IC50 values of 4.07 mg/mL. Cis-platin exhibited the capability of anti-proliferation against Caco-2 cells, with the IC50 value of 35.09 μg/mL. Previously reports revealed many natural polysaccharides possess potent anticancer activity [31–32]. Though the anticancer activity of natural polysaccharides was lower than the activity of synthetic anticancer agents such as Cis-platin, natural polysaccharides possess lower cytotoxicity against common cells than synthetic anticancer agents [18, 32]. Therefore, it is worth to develop these natural polysaccharides as potential anticancer agents, although their anticancer concentrations were usually presented in high levels.

## Conclusions

The response surface methodology proved to be useful for optimizing the extraction process of total sugar from *S*. *thunbergii*, and statistical analysis was a useful and effective tool in selecting optimum extraction conditions. The optimum conditions analyzed by Box–Behnken design showed that liquid:solid ratio of 120 mL/g, extraction time of 210 min, and extraction temperature of 97.29°C were the best conditions. Under the most suitable conditions (A = 120 mL/g, B = 210 min, C = 97°C), the experimental yield of total sugar was 7.53%, which was closed with the predicted yield value 7.85%. In addition, extraction temperature had the significant effects on the yield of total sugar, and we should pay attention to selection of temperature in the isolation of polysaccharides from *S*. *thunbergii*.

The crude polysaccharide from *S*. *thunbergii* contained predominantly water extractable polysaccharide STP-II (63.75%) purified by DEAE Sepharose CL-6B column chromatography. The results of HPSEC with Sugar KS-804 column, GC with HP-5 column and HPLC with Sugar SP0810 column of STP-II indicated that the purified fraction STP-II was homogeneous, had a molecular weight of 550KD, and comprised mainly of fucose, xylose, galactose, glucose and glucuronic acid. On the basis of antioxidant activities assay, STP-II had stronger free-radical-scavenging activities for superoxide radical and hydroxyl radical than Vc. STP-II also showed the noticeable anti-proliferative effect on Caco-2 cells. This research may provide new insights into the development and application of polysaccharides from *S*. *thunbergii*. In order to further provide with theoretical supports for the development and utilization of polysaccharide from *S*. *thunbergii*, the research on the detailed structure of STP-II including the type of glycosidic linkages, morphological information, and the connection type of the protein [33–35] is currently underway in our laboratory.
